# P32 Occurrence of *bla*_KPC_, *bla*_NDM_ and *bla*_OXA-48_ genes among carbapenemase-producing Enterobacteriaceae (CRE) in National Cancer Institute, Sri Lanka

**DOI:** 10.1093/jacamr/dlac004.031

**Published:** 2022-02-16

**Authors:** Y. W. S. Suranadee, H. R. Jyalathacrachchi, S. Gamage, S. P. Gunasekara

**Affiliations:** Department of Medical Microbiology and Immunology, Faculty of Medicine, University of Colombo, Sri Lanka; Department of Medical Microbiology and Immunology, Faculty of Medicine, University of Colombo, Sri Lanka; Department of Medical Microbiology and Immunology, Faculty of Medicine, University of Colombo, Sri Lanka; National Cancer Institute, Sri Lanka

## Abstract

**Background:**

Carbapenemase-producing Enterobacteriaceae (CRE) are a global problem and prevalence of CR genes differs across geographical regions. The current Sri Lankan situation is unknown; hence, this study aimed to identify three commonly occurring CR genes in the region.

**Methods:**

Enterobacteriaceae isolates resistant to carbapenems were collected from clinical samples submitted for routine testing to the microbiology laboratory of National Cancer Institute. Species identification was done using conventional biochemicals and CLSI disc diffusion method was used for ABST of CRE isolates. Identification of CR genes was done by in-house conventional multiplex PCR, using previously described primers (Table 1). Ten microlitres of total DNA from each sample, extracted by heat shock method, was subjected to multiplex PCR in a 50 μL of PCR mixture which contained 5× PCR buffer (10 mmol/L Tris–HCl, 50 mmol/L KCl), 2.5 mmol/L of MgCl2, 10 mmol/L of deoxynucleotide triphosphate, 5 U/mL of Taq Polymerase and 20 μmol/L of each primer. The PCR program was run at 94°C for 3 min, followed by 30 cycles of 95°C for 1 min, 55°C for 30 s and 73°C for 1 min, with a final extension at 72°C for 5 min. Products were visualized by electrophoresis in 2% agarose gel (Figure 1).

**Results:**

A total of 123 isolates were tested. *Klebsiella pneumoniae* was the predominant carbapenemase producer (58.5%). Sixty-six (53.6%) isolates harboured *bla*_OXA-48_ and 47 (38.2%) had *bla*_NDM_. Only one isolate was positive for *bla*_KPC_. Thirteen (10.5%) had both *bla*_OXA-48_ and *bla*_NDM_ and 9 (7%) tested negative for all three genes.

**Conclusions:**

*bla*_OXA-48_ and *bla*_NDM_ being common and *bla*_KPC_ being very rare is expected with the geographical location of the country. Noted high prevalence and co-occurrence of CR genes among CRE isolates is alarming and pose a threat to patient management and infection control of the institution.

**Table 1.** Oligonucleotides used in the study

Primer	Primer (5′–3′)	Gene	Product size
KPC-F	CGTCTAGTTCTGCTGTCTTG	*bla*_KPC_	798 bp
KPC-R	CTTGTCATCCTTGTTAGGCG		
NDM-F	GGTTTGGCGATCTGGTTTTC	*bla*_NDM_	621 bp
NDM-R	CGGAATGGCTCATCACGATC		
OXA48-F	GCGTGGTTAAGGATGAACAC	*bla*_OXA-48_	438 bp
OXA48-R	CATCAAGTTCAACCCAACCG		

**Figure 1.**
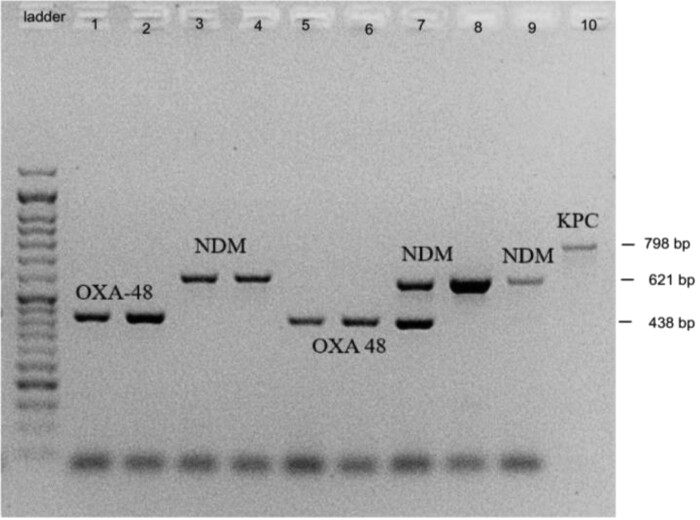
Agarose gel electrophoresis (2%). Samples in lanes 1, 2, 5 and 6 are positive for *bla*_OXA-48_; samples in lanes 3, 4, 8 and 9 are positive for *bla*_NDM_ and sample in lane 10 is positive for *bla*_KPC_. Sample in lane 7 is positive for both *bla*_NDM_ and *bla*_OXA-48_.

